# The Sd^a^ Synthase B4GALNT2 Reduces Malignancy and Stemness in Colon Cancer Cell Lines Independently of Sialyl Lewis X Inhibition

**DOI:** 10.3390/ijms21186558

**Published:** 2020-09-08

**Authors:** Michela Pucci, Inês Gomes Ferreira, Nadia Malagolini, Manuela Ferracin, Fabio Dall’Olio

**Affiliations:** Department of Experimental, Diagnostic and Specialty Medicine (DIMES), General Pathology Building, University of Bologna, Via San Giacomo 14, 40126 Bologna, Italy; michela.pucci3@unibo.it (M.P.); ines.gomesferreira2@gmail.com (I.G.F.); nadia.malagolini@unibo.it (N.M.); manuela.ferracin@unibo.it (M.F.)

**Keywords:** Sd^a^ antigen, sialyl Lewis antigens, transcriptomic analysis, cancer stem cells, non-adherent growth

## Abstract

Background: The Sd^a^ antigen and its biosynthetic enzyme B4GALNT2 are highly expressed in healthy colon but undergo a variable down-regulation in colon cancer. The biosynthesis of the malignancy-associated sialyl Lewis x (sLe^x^) antigen in normal and cancerous colon is mediated by fucosyltransferase 6 (FUT6) and is mutually exclusive from that of Sd^a^. It is thought that the reduced malignancy associated with high B4GALNT2 was due to sLe^x^ inhibition. Methods: We transfected the cell lines SW480 and SW620, derived respectively from a primary tumor and a metastasis of the same patient, with the cDNAs of FUT6 or B4GALNT2, generating cell variants expressing either the sLe^x^ or the Sd^a^ antigens. Transfectants were analyzed for growth in poor adherence, wound healing, stemness and gene expression profile. Results: B4GALNT2/Sd^a^ expression down-regulated all malignancy-associated phenotypes in SW620 but only those associated with stemness in SW480. FUT6/sLe^x^ enhanced some malignancy-associated phenotypes in SW620, but had little effect in SW480. The impact on the transcriptome was stronger for FUT6 than for B4GALNT2 and only partially overlapping between SW480 and SW620. Conclusions: B4GALNT2/Sd^a^ inhibits the stemness-associated malignant phenotype, independently of sLe^x^ inhibition. The impact of glycosyltransferases on the phenotype and the transcriptome is highly cell-line specific.

## 1. Introduction

**Figure 1:**
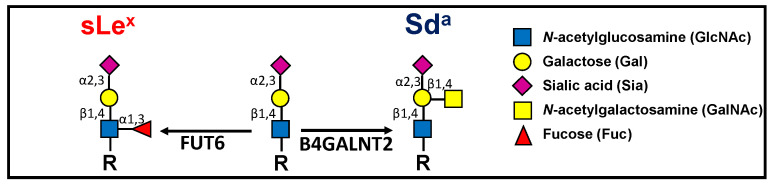
The Sd^a^ and the sLe^x^ antigens derive from alternative and mutually exclusive terminations of a common α2,3-sialylated type 2 structure. The key enzymes for their biosynthesis in colonic tissues are FUT6 and B4GALNT2.

Glycosylation is one of the most prominent modifications of proteins and lipids, consisting of the addition of sugar chains covalently attached to the protein or lipid backbones. Carbohydrate chains play multiple roles, fine tuning the biological interactions between cells and molecules. A variety of carbohydrate structures are altered in cancer and many of them play pivotal roles in shaping the phenotype of the cancer cells and determining the clinical outcome of the disease [1,2,3,4]. Sialyl Lewis^x^ (sLe^x^) is a well-known carbohydrate antigen, formed by a α2,3-sialylated type 2 lactosaminic chain (Siaα2,3Galβ1,4GlcNAc) to which a fucose residue is α1,3-linked to GlcNAc [Siaα2,3Galβ1,4(Fucα1,3)GlcNAc] (Figure 1).

This structure is physiologically involved in leukocyte extravasation, acting as a ligand for cell adhesion molecules of the selectin family. However, it can be also ectopically overexpressed in various neoplasms, including colorectal cancer (CRC), and is responsible for increased malignancy and metastasis [5,6,7,8]. Although the molecular bases of sLe^x^ overexpression in colon cancer are complex and poorly understood [9,10,11,12], its last biosynthetic step in colon is mainly, if not exclusively, catalyzed by fucosyltransferase 6 (FUT6) [13]. The carbohydrate antigen Sd^a^ shares with sLe^x^ a α2,3-sialylated type 2 lactosaminic chain backbone. However, in Sd^a^ an *N*-acetylgalactosamine (GalNAc) residue is β1,4-linked to galactose (Figure 1). The enzyme responsible for Sd^a^ biosynthesis, β1,4-*N*-acetylgalactosaminyltransferase 2 (B4GALNT2) has been first identified in our laboratory [14] and successively cloned in our and other labs [15,16,17]. B4GALNT2 and its cognate antigen Sd^a^ play various important roles in different contexts [18]. The vast majority of Caucasians express the Sd^a^ antigen on erythrocytes and in a few other tissues, although a minority lack Sd^a^ because of an inactivating germline mutation of *B4GALNT2* [19]. At least two different transcripts that only differ in the first exon are generated from the *B4GALNT2* gene. Both transcripts contain a translational start site from which two different transmembrane polypeptides originate: the one referred to as “long form” contains an exceptionally long cytoplasmic tail, while the one referred to as “short form” contains a cytoplasmic domain of conventional length [15,16]. Both forms localize mainly in the Golgi, although the long form exhibits also post-Golgi localizations [20]. B4GALNT2 is expressed at a very high level in normal colon but undergoes a dramatic down-regulation in CRC [21,22,23]. The short form is by far the major type expressed in both normal and cancerous colon [24] and displays higher enzymatic activity than the long form [23]. The molecular bases of the reduced B4GALNT2 expression in colon cancer are not completely clear, although the methylation of the *B4GALNT2* promoter certainly plays a role [25,26]. Transcriptomic data from The Cancer Genome Atlas (TCGA) database indicate that CRC patients with higher B4GALNT2 expression level in cancer tissues display longer overall survival [27]. In gastrointestinal cancer cell lines, it has been shown that the forced expression of B4GALNT2 resulted in a replacement of the sLe^x^ antigen with the Sd^a^ antigen [23,28]. Moreover, in normal colonic tissue B4GALNT2 plays a role in keeping sLe^x^ expression low [24]. The finding that cell lines in which sLe^x^ was replaced by Sd^a^ because of B4GALNT2 expression displayed dramatically reduced malignancy in mouse models [28,29] led to the widely accepted notion that sLe^x^ was the key player of malignancy and that B4GALNT2 played only an ancillary role through the inhibition of sLe^x^ biosynthesis. In this study, we have forced the expression of FUT6 or B4GALNT2 in the CRC cell lines SW480 and SW620. The two cell lines, which were originally devoid of the two enzymes and therefore lacked sLe^x^ and Sd^a^, were generated from the primary tumor (the former) and a lymph node metastasis (the latter) of the same patient. As a result of transfection either with *B4GALNT2* or *FUT6* cDNAs, we established genetic variants of the two cell lines expressing either the sLe^x^ or the Sd^a^ antigen. We found that B4GALNT2/Sd^a^ expression exerts a strong and sLe^x^-independent inhibition of the stemness-associated malignant phenotype, while FUT6/sLe^x^ enhanced some aspects of the malignant phenotype. The impact on the transcriptome induced by the two glycosyltransferases was deep and partially cell type-specific. 

## 2. Results

### 2.1. Transfection of SW480 and SW620 with FUT6 and B4GALNT2 cDNAs

The two colon cancer cell lines were chosen as recipients of FUT6 and B4GALNT2 enzymes for two reasons. First, because they lacked both enzyme activities [13]. Second, they were derived from the primary tumor (SW480) and a lymph node metastasis (SW620) of the same patient [30], thus allowing to investigate the effect of the two enzymes at different tumor stages. As shown in Figure S1A, in both SW480 and SW620 cell lines *B4GALNT2*-transfection induced the expression of the Sd^a^ antigen, whereas introduction of FUT6 plasmid led to sLe^x^ expression. Mock transfectants exhibited negligible levels of the two antigens. The mRNA level of the two glycosyltransferases, as determined by microarray analysis, was negligible in mock transfectants but well expressed in the respective glycosyltransferase transfectants (Figure S1B). While in FUT6 transfectants the level of FUT6 mRNA was very high, in B4GALNT2 transfectants the level of B4GALNT2 mRNA was unexpectedly low, although more than sufficient to ensure a good level of Sd^a^ antigen expression. 

### 2.2. Phenotypic Changes Induced by B4GALNT2 and FUT6 Expression

To unravel the relative contribution of the Sd^a^ and sLe^x^ antigens to the phenotype of the two cell lines, the following aspects of malignant growth were analyzed *in vitro*.

**Figure 2:**
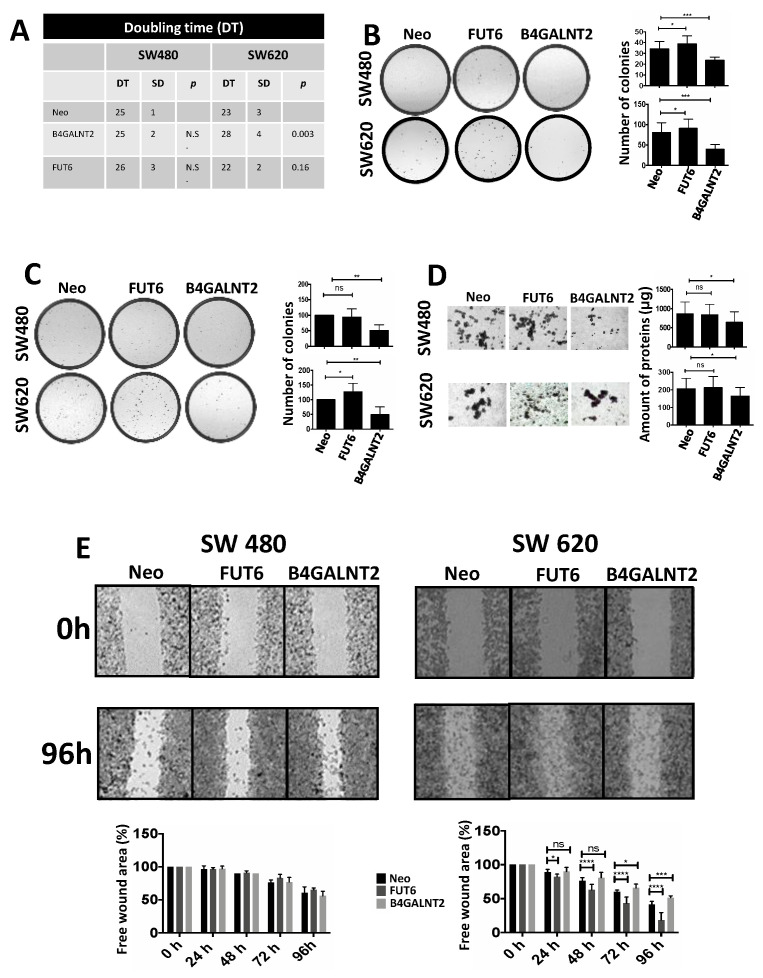
Phenotypic effects induced by FUT6 or B4GALNT2 expression. (**A**) The doubling time (DT), expressed in hours, was calculated as described in Materials and Methods in at least three independent experiments performed in duplicate. (**B**) Fifty cells of the different populations were seeded in a 6-well plate in standard conditions of growth. After 15 days, the colonies were fixed, stained and counted. Histograms indicate the total number of colonies. The assay was repeated 8 times. (**C**) Ten thousand cells of the different populations were seeded in 6-well plates in 0.33% agar. After 3 weeks, the colonies were fixed, stained, photographed without magnification and the colonies visible to the naked-eye were counted. (**D**) Each well of a 6-well plate was coated with a 0.5% agar solution in complete L-15 medium to avoid adhesion of the cells to the plastic surface. Ten thousand cells were then seeded in complete L-15 medium. The aspect of the spheroids is shown, but the photographs are not necessarily representative of their amount. To quantify the number of cells grown as spheroids or single cells in liquid phase, the cells were quantitatively collected, pelleted by centrifugation and homogenized. The total amount of protein was calculated and taken as a measure of the cells grown. (**E**) The healing of a wound in a monolayer of confluent cells was monitored every 24 h. The free area of the wound was quantitated by ImageJ and normalized to the free area of the same cell line at 0 h, which was taken as 100%. The photographs show only the start (0 h) and the end point (96 h) of the healing process. Graphs in the bottom report the quantification of the healing process at each time point. The microphotographs were taken at a 4× magnification. In (**A**–**E**) statistical analysis was performed using one-way analysis of variance (ANOVA) and Dunnett’s multiple comparisons test. ns, not significant; * *p* ≤ 0.05; ** *p* ≤ 0.01; *** *p* ≤ 0.001; **** *p* ≤ 0.0001.

**Figure 3:**
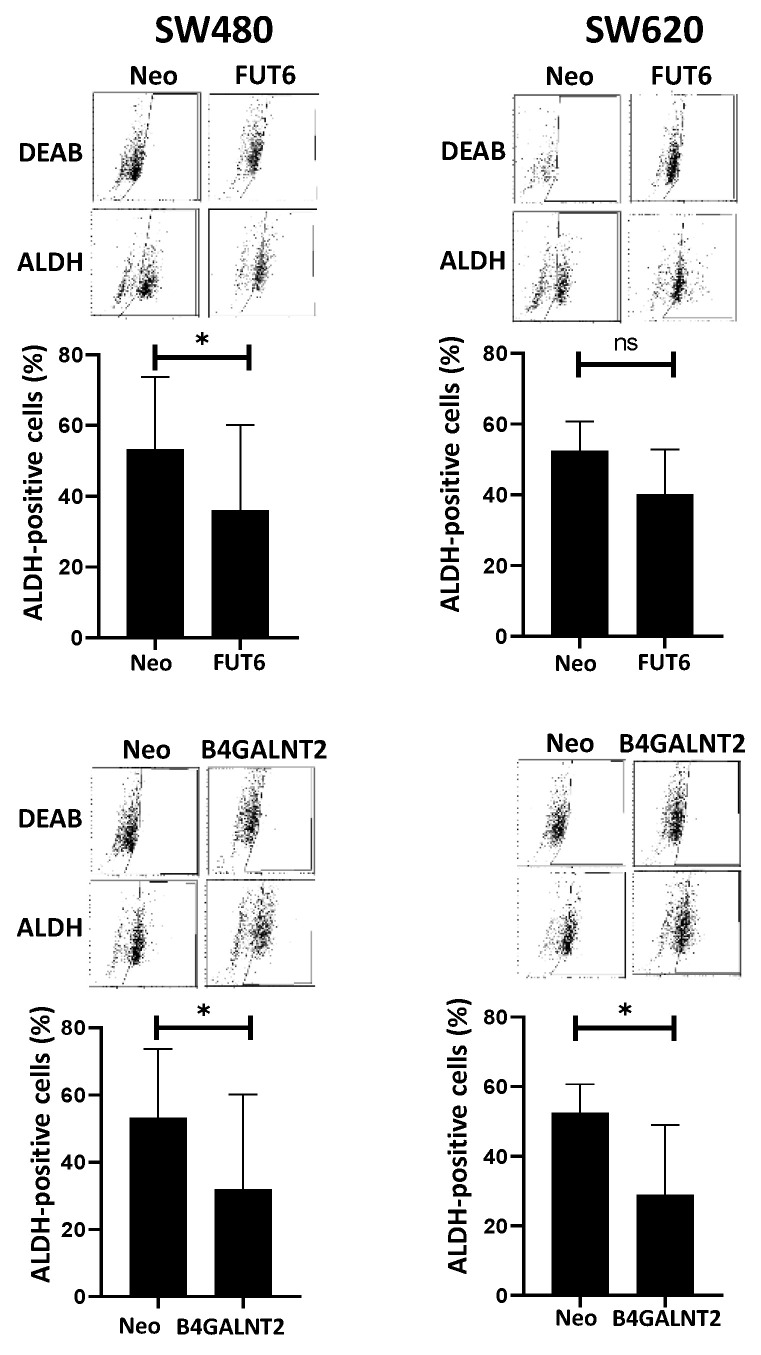
ALDEFLUOR analysis. Cells were incubated with ALDEFLUOR either in the presence or in the absence of the inhibitor N,*N*-diethylaminobenzaldehyde (DEAB). Gates excluding all of the cells labelled in the presence of DEAB were set. Cells included in the gate in the absence of DEAB, were considered to be aldheyde dehydrogenase 1 (ALDH)-positive. Histograms report the percentage of ALDH positive cells ±SD in five independent experiments. * *p* ≤ 0.05.

- Doubling time. Expression of B4GALNT2 reduced the speed of growth of SW620, increasing the doubling time from 23 ± 3 to 28 ± 4 h (Figure 2A). Although a tendency to increased speed of growth in FUT6-expressing SW620 cells was observed, it did not reach statistical significance. On the other hand, no effect of either glycosyltransferase was observed on the doubling time of SW480.
- Clonogenic ability. The number of cells able to form colonies was affected by both glycosyltransferases in both cell lines (Figure 2B). The cell line SW620 was provided with a higher capability to form colonies, compared to SW480. However, in both cell lines FUT6 expression induced a small but significant increase of the clonogenic ability which, by contrast, was strongly impaired by B4GALNT2.
- Soft agar growth. In both cell lines, B4GALNT2 expression significantly reduced the formation of clones, while FUT6 induced a slight increase of clone formation only in SW620 cells (Figure 2C).
- Spheroid formation. This assay measures the ability of the cells to survive and proliferate in liquid medium, a condition associated with stemness, even more drastic than soft agar. SW480 cells formed mainly rounded spheroids with regular edges, whereas SW620 cells formed spheroids with irregular shape and many cells were found as single. B4GALNT2 expression reduced the formation of spheroids in both cell lines while FUT6 had no effect (Figure 2D). Owing to the fact that floating particles were nearly impossible to quantify, the growth of spheroids was indirectly quantified by collecting all the floating particles, preparing a homogenate and calculating the total amount of protein of the homogenate.
- Wound healing assay. The ability to heal a wound in a layer of confluent cells provided an example of the differential response of the two cell lines to glycosyltransferase expression. In fact, in the cell line SW480 the expression of either FUT6 or B4GALNT2 left unaltered the ability to heal a wound. On the contrary, in the cell line SW620 the healing capability was greatly enhanced by FUT6 but reduced by B4GALNT2 (Figure 2E).
- ALDEFLUOR assay. This assay for stemness revealed that B4GALNT2 induced a marked down-regulation of the number of stem cells in both cell lines (Figure 3). Unexpectedly, FUT6 induced a slight reduction of aldheyde dehydrogenase 1 (ALDH)-positive cells, which reached statistical significance only in SW480.

### 2.3. Effect of B4GALNT2 or FUT6 Expression on the Transcriptome

**Figure 4:**
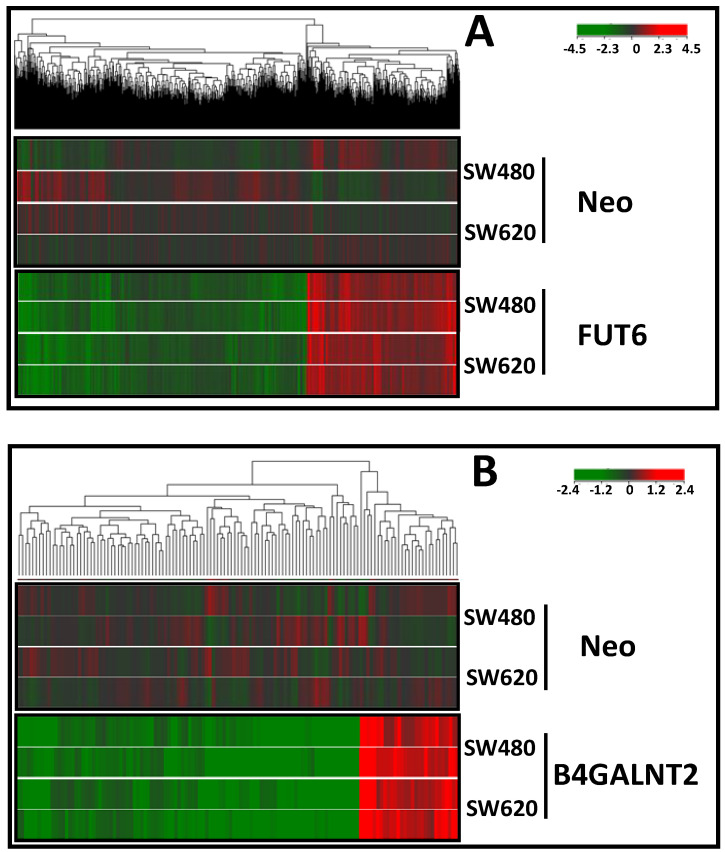
Heatmaps of genes modulated upon FUT6 and B4GALNT2 expression. (**A**) Cluster analysis of SW480 and SW620 cells transfected with FUT6 and compared with control Neo. (**B**) Cluster analysis of SW480 and SW620 cells transfected with B4GALNT2 and compared with control Neo. Differentially expressed genes are reported. Genes (columns) and samples (rows) were grouped by hierarchical clustering (Manhattan correlation). High- and low-expression was normalized to the average expression across all samples. Differences were analyzed by the moderated t-test. Corrected *p*-value cut-off: 0.05; multiple test correction used: Benjamini–Hochberg. Color codes refer to the level of up- or down-regulation.

We analyzed the effect of *B4GALNT2* and *FUT6* transfection on the global gene expression of SW480 and SW620 cell lines. We identified genes significantly and consistently modulated either by FUT6 or B4GALNT2 in both SW480 and SW620 cells. The heat maps of the genes modulated in SW480 and SW620 transfected either with B4GALNT2 or FUT6 or mock-transfected (Neo) are shown in Figure 4. The impact on the transcriptome of the two glycosyltransferases was assessed by Pathway Enrichment analysis and found to be very different. In fact, FUT6 modulated 1779 genes while B4GALNT2 modulated only 128 genes. This resulted in a very different predicted impact on the cell behavior (Table 1 and Table 2).

Both glycosyltransferases appeared to impact cell cycle (5 networks by FUT6 and 1 by B4GALNT2), cytoskeleton (1 network by FUT6 and 3 by B4GALNT2), and cell adhesion (1 pathway each). The pathway “Development neurogenesis-axonal guidance” was affected by both glycosyltransferases, although through different genes. In the context of colon cancer cells, it probably reflects the ability to migrate.

To obtain more detailed information on the possible phenotypic effects of the expression of the two glycosyltransferases, we performed an analysis restricted to the genes showing a level of expression either in Neo or in glycosyltransferase-transfected cells ≥ 50 and a fold change ≥ 2 (for B4GALNT2) or ≥ 3 (for FUT6, owing to the much greater number of modulated genes) (Supplementary Tables S1 and S2). The main role of each gene was deduced from the GeneCards web site (https://www.genecards.org/). Out of the 63 FUT6-modulated genes reported in Table S1, 10 displayed up-regulation, whereas 53 were down-regulated. Among the 45 genes included in Table S2, 11 showed up-regulation while 34 displayed down-regulation in B4GALNT2 expressing SW480 and SW620 cells. Besides these genes presenting a parallel modulation in the two cell lines, another group underwent a glycosyltransferase-induced modulation in either cell line but not in both (data not shown). The genes consistently modulated in both cell lines, as well as those showing modulation in either cell line, were classified in broad functional categories (Table 3 and Table 4). Expression of FUT6 affected several functional classes in both cell lines, in particular, “Transcription”, “Cell adhesion” and “Signal transduction” (Table 3), but appeared to be cell-type specific. In SW620, the effect of FUT6 expression was very strong and impacted groups of genes poorly or unaffected in SW480, specifically a cluster of genes directly involved in DNA duplication, such as the telomerase reverse transcriptase (*TERT*), thymidylate synthase (*TYMS*), the component of the DNA polymerase Ɛ-complex (*POLE4*), and the origin recognition complex member *ORC6*. Yet, genes related to “Cytoskeleton-cytokinesis”, “Growth factor receptors”, “Ion transport”, “Signal transduction” and “Transcription” appeared to be more deeply affected by FUT6 in SW620 than in SW480. Surprisingly, on a few genes such as *CYB5R2*, *DNAJC15*, *MVB12B* and *LIPC* (marked in bold), FUT6 induced opposite regulation in the two cell lines. 

Among the functional classes more widely affected by B4GALNT2 in both cell lines we found “Apoptosis”, “Cytoskeleton and cytokinesis” and “Transcription” (Table 4). The response to B4GALNT2 was also strongly cell line-specific. In fact, an additional large group of genes belonging to the “Cytoskeleton and cytokinesis” class was modulated only in SW620, while a higher number of genes belonging to the “Transcription” and “Growth factors” categories were modulated by B4GALNT2 only in SW480 cells. Noteworthy, six SNAR [Small NF90 (ILF3) Associated RNAs], a group of non-protein coding RNAs with an unclear role in RNA function, were up-regulated only in SW480 cells. 

Unexpectedly, we observed that transfection with either glycosyltransferase gene induced consistent gene modulation in one of the two cell lines (Table S3). For example, the gene *ANLN*, which encodes for an actin-binding protein required for cytokinesis, was up-regulated in SW620 by expression of either B4GALNT2 or FUT6. The ten genes belong to different functional classes and the biological significance of this phenomenon remains to be established.

Owing to the marked cell specificity of the effects of glycosyltransferase overexpression we observed in SW480 and SW620 cells, we sought to compare the effects of B4GALNT2 expression in SW480 and SW620 with those previously observed in the B4GALNT2-transfected cell line LS174T [27]. Statistical analysis was thus performed in search of a “B4GALNT2 signature”, common to the three cell lines. Amongst the seven protein coding genes identified by the analysis (Table 5), only *MCOLN2* displayed up-regulation, while the remaining were down-regulated. No information was available on the role of *PLLP* and *FAM321A* in cancer. Following the literature, a tumor-promoting activity for the up-regulation of *MCOLN2* and the down-regulation of *FILIP1L* and *BCL2L10* can be hypothesized. In contrast, the down-regulation of *SPON2* and *COL20A1* would exert a tumor-restraining activity. However, except for *SPON2*, the level of expression of the other genes was often so low that they can hardly be responsible for relevant biological effects in all the three cell lines. 

## 3. Discussion

In this study we have investigated the effect of the expression of FUT6 and B4GALNT2 on two CRC cell lines. These two enzymes can be considered alternative not only because they enzymatically compete for the same acceptor, generating two alternative structures, but also for the association of the first with high malignancy and the second with low malignancy. The two cell lines used to express the two enzymes are closely related, being derived from the primary tumor and a metastasis of the same patient. This allowed to evaluate the effect of the two glycosyltransferases in two cell lines with different malignancy but with a very close genetic background. In agreement with our previous results on LS174T cells [27], we found that in both SW480 and SW620 cells B4GALNT2 attenuated *in vitro* malignancy and stemness. However, the effect on the single properties was very cell-type specific. In fact, in SW620 all the six studied properties were down-regulated by B4GALNT2 whereas in SW480 cells the proliferation rate and the wound healing ability were not affected (Table 6). The only features which appeared to be consistently inhibited by B4GALNT2 in all three cell lines (including LS174T) were the ability to grow in poor adherence and ALDH expression, all related with stemness (Table 6). Importantly, current results indicate that these B4GALNT2-induced effects were totally independent of sLe^x^ inhibition, since they were present even in cell lines lacking sLe^x^. Considering that the two cell lines were derived from the same person and share the majority of the genetic background, it appears that B4GALNT2 is able to inhibit specifically those properties, such as increased growth rate and ability to heal a wound, which are more pronounced in SW620 because of their metastatic origin. It can be hypothesized that in SW620 cell proliferation is fuelled by additional transduction pathways, which are specifically inhibited by B4GALNT2. Numerous experimental studies indicated that the expression of FUT6/sLe^x^ positively impacts malignancy of CRC cells by modulating migration, proliferation and tumor formation [31,32,33,34]. In our system, FUT6/sLe^x^ overexpression increased the number of colonies formed in both cell lines in standard growth conditions. However, in SW620 cells (but not in SW480) FUT6/sLe^x^ increased also the soft agar growth and the wound healing capability. This last observation is remarkable because the intrinsic ability to migrate and colonize the wound from the interior, which is displayed by SW620 Neo, is boosted by FUT6. Apparently, the effect of FUT6 on migration ability could be exerted only if this ability was already present (like in SW620) but it could not be induced *de novo* if not present (like in SW480). Transcriptomic analysis revealed that FUT6 modulated a much higher number of genes than B4GALNT2 in both cell lines. Both glycosyltransferases impacted networks regulating the cell cycle and the cytoskeleton, although through different genes, suggesting that the induced biological effects can be different and even opposite. A closer look at the more prominently expressed and regulated genes showed that, besides many genes regulated in parallel in the two cell lines, others were modulated only in one of the two. Among the genes more consistently down-regulated in both cell lines by B4GALNT2 is *CD44*. Considering the reported function of CD44 as a sLe^x^ carrier [35], and the role of fucosylated CD44 in CRC progression [34], current data provide a new potential mechanism through which B4GALNT2 hinders FUT6-mediated cancer progression; not only through the well documented competition for the carbohydrate substrate acceptor, but also through the down-regulation of one of the preferred sLe^x^ carriers. Down-regulation of CD44, which is a well-known stemness marker, is consistent with the reduced stemness induced by B4GALNT2 expression.

We observed that a small but relevant number of genes belonging to different functional classes were consistently modulated by either glycosyltransferase in the same cell line. As a tentative explanation for this unexpected observation, we propose that the presence of either the sLe^x^ or the Sd^a^ antigens on the same glycan receptor can, in some cases, produce a similar biological effect (for example the binding inhibition of a soluble factor).

While the number of genes consistently modulated by B4GALNT2 in the related cell lines SW480 and SW620 was high, the search for a “B4GALNT2 signature” in common with the unrelated cell line LS174T produced a list of only seven genes. Among these, the down-regulation of *SPON2*, which encodes spondin2, an extracellular matrix protein promoting cell motility, growth and invasion of CRC [36], could explain some of the phenotypic effects induced by B4GALNT2 in the three cell lines. Although high *SPON2* expression is associated with shorter patient survival, no relationship exists between *B4GALNT2* and *SPON2* expression in TCGA data (data not shown). 

Our current and previous data indicate that glycosyltransferases may induce a given phenotypic trait through the activation of multiple interconnected and converging pathways that are activated by different gene expression signatures in a strongly cell-specific manner. One of the main limitations of this study is in the identification and elucidation of these pathways which, at this stage, is still preliminary. 

In the clinic, the level of B4GALNT2 expressed by CRC tissues can be useful to stratify patients according to their risk of progression. This goal is very important to spare low risk patients from invasive and expensive therapies. In this light, it is crucial to understand the mechanism(s) through which B4GALNT2/Sd^a^ exert their tumor restraining activity. If this activity was exerted only through sLe^x^ inhibition, one should expect that it was not working in sLe^x^-negative cases. This work provides evidence that this is not the case because the tumor restraining effect of B4GALNT2 in CRC is largely independent of sLe^x^ inhibition but is due to intimate changes of the colon cancer cell biology. 

## 4. Materials and Methods 

### 4.1. Cell Lines

The cell lines SW480 (ATCC^®^ CCL-228™) and SW620 (ATCC^®^ CCL-227™) were derived from the primary colorectal adenocarcinoma of a 50 year old Caucasian man, Dukes stage B, and from its lymph node metastasis, respectively [30]. Cells were cultured in Leibovitz’s L-15 Medium supplemented with 10% fetal bovine serum (FBS), 1% L-Glutamine and 1% Penicillin/Streptomycin at 37 °C in the absence of CO_2_ in a humidified incubator.

*FUT6* transfection was performed with a *FUT6* pcDNA.1 expression plasmid and an empty pcDNA3.1 for G418 resistance in a 10:1 ratio, as previously described [13]. *B4GALNT2* transfection was performed with the cDNA of the short form of *B4GALNT2* cloned in pcDNA.3 as detailed previously [23]. Mock transfections with empty pcDNA.3 to obtain negative control Neo transfectants were set in parallel. Cells were selected with 1mg/mL G418. G418-resistant polyclonal populations, were cloned. Single clones were isolated, expanded and screened for the expression of the Sd^a^ or sLe^x^ antigens. For both SW480 and SW620, the following cell populations were used: SW480 or SW620 Neo, which are a negative control formed by mock-transfected, G418-resistant cells; SW480 or SW620 FUT6, which are a pool of three clones, highly expressing FUT6 and sLe^x^ antigen; SW480 or SW620 B4GALNT2, which are a pool of three clones, highly expressing B4GALNT2 and Sd^a^ antigen. 

### 4.2. Slot Blot Analysis of Carbohydrate Antigens

Cells were collected by trypsinization and homogenized in ice cold water. The protein concentration of the homogenates was determined using the Lowry method. Thirty micrograms of protein homogenates were spotted on a nitrocellulose membrane in a final volume of 100 μL using a slot- blot apparatus. Incubation was performed as previously described using the anti-Sd^a^ KM694 antibody, kindly provided by Kyowa Hakko Kogyo Co. Ltd., Tokyo, Japan, diluted 1:2000 or the anti sLe^x^ antibody CSLEX1 (ATCC HB85-80) diluted 1:500. As secondary antibodies, anti IgM or anti IgG diluted 1:10,000 were used. The reaction was revealed using Westar ηC 2.0 from Cyanagen, according to the manufacturer’s instructions and detected by autoradiography.

### 4.3. Doubling Time Assay

In 6-well plates, aliquots of 2 × 10^5^ cells for well were seeded in duplicate. After 24 h incubation at 37 °C, the cells of 2 wells were harvested and counted. This number of cells was considered T0. Pairs of wells were harvested and counted 48 h later (T48). The doubling time (DT) was calculated by using the following formula: DT = (48) × 0.3/Log(N°cells_T48_/N°cells_T0_). Statistical analysis was performed by using one-way analysis of variance (ANOVA) and Dunnett’s multiple comparisons test.

### 4.4. Clonogenic Assay

About 50 cells diluted in 2 mL of complete L-15 medium were seeded in triplicate in 6-well plates and incubated at 37 °C. After 15 days, the plates were washed with phosphate buffered saline (PBS) and the colonies were fixed and stained for one hour at room temperature with a solution containing formaldehyde 4% and Crystal Violet 0.005% in PBS. Photographs of the wells were taken without magnification and the colonies visible at naked-eye were counted. Statistical analysis was performed using one-way ANOVA and Dunnett’s multiple comparisons test.

### 4.5. Soft Agar Assay

Each well of 6-well plates was coated with a 0.5% agar solution in complete L-15 medium. On the top of this layer, we distributed a 0.3% agar solution in complete L-15 medium, containing 10,000 cells per well in six replicas. The plates were incubated at 37 °C for 3 weeks, fixed and stained for one hour with a solution containing 4% formaldehyde and 0.005% Crystal Violet in PBS. Photographs of the wells were taken without magnification and the colonies visible to the naked-eye were counted. Statistical analysis was performed using one-way ANOVA and Dunnett’s multiple comparisons test.

### 4.6. Spheroid Assay

Each well of 6-well plates was coated with a 0.5% agar solution in complete L-15 medium as above. On the top of this layer, we seeded about 10,000 cells in complete L-15 medium in six replicas. The growth of the cells as spheroids in liquid medium was monitored every 2–3 days. To quantify the amount of cells grown as spheroids in liquid phase, the cells were quantitatively collected, pelleted by centrifugation and homogenized. The protein concentration of the homogenate was assessed by Lowry assay and its volume was measured. The total amount of protein was calculated and taken as a measure of the cells grown. The statistical analysis was performed using one-way ANOVA and Dunnett’s multiple comparisons test. 

### 4.7. Wound Healing Assay 

The wound healing assay was performed using Culture-Insert 2 Well (Ibidi, Grafelfing, Germany) as previously described [37]. Aliquots of 7 × 10^5^ cells were seeded in each well. When the cells reached confluency, the insert was removed and healing of the wound was measured by taking pictures at 4× magnification. The area free of cells was measured using the MRI Wound Healing Tool in ImageJ (http://dev.mri.cnrs.fr/projects/imagej-macros/wiki/ Wound_Healing_Tool). Statistical analysis was performed using one-way ANOVA and Dunnett’s multiple comparisons test.

### 4.8. ALDEFLUOR Assay

This assay was performed as previously described [27]. Briefly, ALDEFLUOR (Stem Cell technologies, Vancouver, BC, Canada) was added to 5 × 10^5^ aliquots of cells. Half of the cell suspension was treated with DEAB, a specific ALDH inhibitor to set the negative control. Cells were analyzed by flow cytometry and the DEAB-treated sample was used to set a gate excluding all the cells. Non-DEAB-treated cells included in this area were considered ALDEFLUOR positive.

### 4.9. Transcriptomic Analysis 

Transcriptomic analysis of RNA from SW480 and SW620 cells transfected with FUT6 or B4GALNT2 and their respective Neo negative controls was performed in duplicate using Agilent whole human genome oligo microarray (G4851A) as described [38]. Statistical analysis was performed using moderated t-test and the false discovery rate was controlled with the multiple testing correction Benjamini–Hochberg with Q = 0.05. Pathway analysis of differentially expressed genes was determined using the web-based software MetaCore (GeneGo, Thomson Reuters). Gene function was studied through an extensive literature search.

## Figures and Tables

**Table 1:** Networks modulated by FUT6 expression.

Network	*p*-Value	Network Objects
Cell cycle-Mitosis	4 × 10^−7^	Cyclin B1, Cyclin B, Cyclin B2, Histone H3, PBK, Cyclin A, PLK1, Securin, CENP-H, SIL, Separase, HZwint-1, CENP-F, CAP-G/G2, Aurora-A, CDC25, CDC25C, Tubulin-β, KNSL1, CAP-E, AF15q14, HEC, CENP-E, TPX2, SPBC25, ASPM, MAD2a, Survivin, BUB1, CAP-C, Actin, Histone H1, CENP-A
Cell cycle-Core	7 × 10^−7^	CDC45L, Cyclin B1, Cyclin B, Cyclin B2, Cyclin A, PLK1, Securin, RPA3, CENP-H, Separase, CAP-G, Aurora-A, CDC25C, CAP-E, p18, HEC, p21, CENP-E, ORC6L, CKS2, MAD2a, Survivin, BUB1, CAP-C, CENP-A
Cytoskeleton-Spindle microtubules	2 × 10^−6^	Cyclin B1, Cyclin B, Cyclin B2, KIF4A, DEEPEST, PLK1, Securin, CENP-H, GTSE1, Separase, HZwint-1, CENP-F, Aurora-A, Tubulin-β, KNSL1, Tau (MAPT), HEC, MKLP2, CENP-E, CKS2, MAD2a, BUB1, CENP-A
Development-Regulation of angiogenesis	2.5 × 10^−6^	MMP-9, FOXM1, IL-8, PKC, CD13, Oct-3/4, TRIP6, TrkB, GLI-1, WT1, DBH, Cathepsin B, Ephrin-B, Ephrin-A, PLC-β, Gα(i)-specific peptide GPCRs, c-Myc, IL8RB, Gα(q)-specific peptide GPCRs, PI3K reg class IA, STAT5, IL-15, Ephrin-A receptors, p21, Plasminogen, Angiostatin, Plasmin, Ephrin-B receptor 4, Ephrin-B receptors, IP3 receptor, IL-1RI, Ihh, Hedgehog, EGFR, PLAUR (uPAR), EDNRB
Cell cycle-G2-M	1 × 10^−5^	FOXM1, Cyclin B1, Cyclin B, Cyclin B2, Histone H3, MYRL2, MRLC, Cyclin A, Cyclin A2, PLK1, Securin, GTSE1, Claspin, CAP-G, CAP-G/G2, Aurora-A, CDC25, CDC25C, RGC32, KNSL1, CAP-E, c-Myc, p21, Rad51, BLM, CKS2, MAD2a, BUB1, EGFR, CAP-C, Histone H1.5, Histone H1, FANCD2
Cell cycle-S phase	5 × 10^−5^	CDC45L, Cyclin B1, Cyclin B, Cyclin B2, Histone H3, Cyclin A, Cyclin A2, Histone H4, PLK1, Securin, RPA3, Separase, DRF1, PDS5, RGC32, PRIM2A, p21, ORC6L, Rad51, AHR, DDX11, BUB1, Histone H1.5, Histone H1, Sgo1
Cell cycle-Meiosis	7 × 10^−5^	Cyclin B1, HSP70, Cyclin A, GCNF, PARD3, PLK1, Securin, SMC1L2, FANCG, RAD54L, Separase, CDC25C, Tubulin-β, c-Myc, PP2A regulatory, PI3K reg class IA, Rad51, BLM, RAD54B, EGFR
Development-Neurogenesis-Synaptogenesis	5 × 10^−4^	FGF7, APOE, Syntaxin 1A, TrkB, ErbB3, nAChR alpha, WNT, Ephrin-B3, Ephrin-B, Neurexin beta, Ionotropic glutamate receptor, Kainate receptor, NT-4/5, Neuregulin 2, NMDA receptor, Frizzled, MAGI-1(BAIAP1), FGFR2, Synaptotagmin VII, Synaptotagmin, Ephrin-B receptors, X11, FGFR4, Endophilin A3, Actin, NR1
Cell adhesion-Attractive and repulsive receptors	7 × 10^−4^	5T4, Semaphorin 3A, MENA, SLIT1, c-Fes, UNC5B, AF-6, Ephrin-B3, Ephrin-B, Ephrin-A, Ephrin-A3, Ephexin, Tau (MAPT), L1CAM, PI3K reg class IA (p55-gamma), PI3K reg class IA, Ephrin-A receptors, Ephrin-A receptor 3, Collagen XIII, Ephrin-B receptor 4, Ephrin-B receptors, RHO6, Actin, Integrin, Intersectin
Development-Neurogenesis-Axonal guidance	1 × 10^−3^	AHNAK, Syntenin 2, APOE, Semaphorin 3A, PKA-reg (cAMP-dependent), PARD3, CRMP4, TrkB, MENA, SLIT1, c-Fes, Ryanodine receptor 1, UNC5B, Ephrin-B3, Ephrin-B, Ephrin-A, Ephrin-A3, PLC-β, NT-4/5, L1CAM, PI3K reg class IA, Ephrin-A receptors, Ephrin-A receptor 3, Guanine deaminase, Ephrin-B receptor 4, Ephrin-B receptors, RHO6, IP3 receptor, Actin, Integrin

**Table 2:** Networks modulated by B4GALNT2 expression.

Network	*p*-Value	Network Objects
Cell adhesion- Cell matrix interactions	0.002	Mindin, Galectin-7, CD44 (EXT), CD44 (ICD), CD44 soluble, CD44
Cell cycle-S phase	0.012	Rad51, MCM10, ORC6L, RGC32
Development-Neurogenesis-Axon guidance	0.012	DISC1, Mindin, Semaphorin 3B, PLC-β, Netrin-1
Cytoskeleton-Intermediate filaments	0.013	Tubulin-β2, Tubulin-β, Kinesin heavy chain
Reproduction-GnRH signaling pathway	0.017	mGluR8, Gα(i)-specific metabotropic glutamate GPCRs, PLC-β, PLC-β1
Cytoskeleton-Regulation of cytoskeleton rearrangement	0.024	SPTBN(spectrin1-4), Tubulin-β2, Tubulin-β, CD44
Reproduction-Gonadotropin regulation	0.031	mGluR8, Gα(i)-specific metabotropic glutamate GPCRs, PLC-β, PLC-β1
Cytoskeleton-Cytoplasmic microtubules	0.032	Tubulin-β, Kinesin heavy chain, KIF5A
Reproduction-Feeding and Neurohormone signaling	0.037	CD44, PLC-β, PLC-β1, AKR1C1

**Table 3:** FUT6-modulated genes in either SW480 or SW620 or in both, grouped for functional classes.

Functional Class	Both in SW480 and SW620	Only in SW480	Only in SW620
Apoptosis	*BEX2; PTPN13; HRK*		*RSL1D1; BIRC3; PPM1K*
Ca binding	*CALB2*		*CAB39L*
Cell adhesion	*MCAM; GPR126; ANTXR2; SLIT1; PEAR1; NTN4*	*ITGB7; NEBL*	*CDH16;* *DOCK4; AGR2*
Cell cycle	*BRSK2; BEX2*	*CCNI*	*TERT; ORC6; CDKN2C; TYMS; POLE4*
Chromatin remodelling		*HIST1H2AI; HIST1H2BE; HIST1H2AG; HIST1H1B; HIST1H4L*	
Cytoskeleton-cytokinesis	*MYH7B; CENPI; * *BRSK2; TUBB2; FILIP1*	*LLGL2; FGFR1OP; WDR1;* *RGCC; CEP95; TUBB2A*	*TRIM58; FRMD4A; ANLN; TNNC1; KIF18A; DNM3; MICAL3;* *APC2; MARCKS; KIF19; TUBB2B*
DNA damage response			*RAD51AP1*
Drug metabolism		***CYB5R2***	***CYB5R2**;* *CYP2J2; ADH1C*
Energy production		***DNAJC15***	***DNAJC15***
Extracellular matrix	*SCEL; HS3ST1*	*COL9A3; COL6A1*	*FMOD; SDC4*
Glycosylation			*GALNT18*
Growth factors	*MIA*	*IGFBP2;* *MDK*	*IHH; KITLG;* *WLS*
Growth factors receptors	*GRB10; GPR126; FZD6; CALCA*	*NTRK2*	*GFRA3;* *EFNB3; GPR160; NOTCH2; RAMP1; SMO*
Hypoxia response	*HEPAS1*	*HIF1A*	
Inflammation and immunity	*CD55; CXCL8; NCF2*	*ANXA1*	*RARRES2*
Intracellular transport	*CPLX2; * *CPLX1; CAPN8*	***MVB12B**;* * SEZ6L2; GOLGA8A*	*RAB36; HIP1; **MVB12B**; * *BLOC1S4; HTT; CPE; AGR2; SPIRE2*
Ion transport	*KCNJ2; * *AHNAK2; TRPV6; BEST1; * *FXYD4*		*TMC4; AKAP7; PIEZO1; MFSD10; BSPRY; MTL5; CACNA2D4; ATP6V0A4*
Lipid metabolism		***LIPC; CYB5R2***	*PLIN4;**LIPC**;** CYB5R2**; CYP2J2*
Mucosa protection		*TFF1; TFF3; SLPI*	*AGR2*
Nuclear structure and function		*NPIPB5*	*NOP14*
Phosphatases		*SGPP2*	*DUSP23; PPM1K*
Proteolysis	*SERPINE2; TIMP3;* * TPSAB1; TFPI*		*MME;* * WFDC2*
RNA maturation	*GPAT2; SNORA30; SNORA62*	*SNORA75; SNORA2B; SNORA13; SFPQ; CLK1*	*TRA2A*
Signal transduction	*RASGEF1A; CAPN5; AKAP12; GBP3; ADRBK2; REPS2*	*AFAP1L2; SGPP2; * *NGEF; PLCB1; CRABP2; PIM1; PTPRM; PTPRS*	*CHN2; SHCBP1; GPER1; CNPY1; * *APC2; DGKQ; MYZAP; NOTCH2NL; SQSTM1; LMTK3; ARHGEF4; PROM1; PKIB; TSPAN5; RRAGD; PTPN13; GRB10; AKT3*
Stress response	*OXR1*		
Transcription	*CAPN15;* * BEX2; BHLHE41; DIP2C; HEPAS1; CREB5; ZNF462; HES7; ZIC5*	*LEF1; PAX6; * *HIF1A; RNF187; TCEA3; TCEA2*	*PRRX1; KLF17; * *NELFA; ZNF581; MXD4; RCOR2; MXI1; ESSRA; MYCL; RELB; FOXD1; BCL3; ZNF22*
Translation		*EEF1A2*	
Transporters		*SLC43A3;* * ABCC3*	*SLC39A11; SLC29A2*
Ubiquitin proteasome pathway		*UBASH3B;* * OTUD1*	*NEURL3*

Only genes showing a fold change “Mean FUT6/Mean Neo” ≥3, an adjusted *p* value ≤ 0.05 and a level of expression either in Neo or in B4GALNT2 ≥ 50 are reported. Genes up-regulated or down-regulated are marked in red or blue, respectively. Genes showing opposite regulation in the two cell lines are in bold.

**Table 4:** B4GALNT2-modulated genes in either SW480 or SW620 or in both, grouped for functional classes.

Functional Class	Both in SW480 and SW620	Only in SW480	Only in SW620
Apoptosis	*G0S2;* * CDIP1; LGALS7; RNF157; FILIP1L; SEMA3B*	*RNF130*	*EVA1A; PTPN13; CDH13*
Cell adhesion	*CD44; CDHR2; DDR1*	*ELSPBP1; * *PCDH9*	*NEBL; SERPINB8*
Cell cycle	*ORC6;* * DISC1; RGCC*		*CDK14;* * BRSK2*
Chromatin remodelling	*MTA2*	*ING4; BAZ2B*	*ATRX;* * ZNF462*
Cytoskeleton-cytokinesis	*ORC6; CDCA5; MCM10; * *DISC1; SPIRE2; PTPRN2; SEMA3B; TUBB2A; KRT15; SPTBN5*	*DLC1;* * SHROOM2*	*CENPI; ASPM; ERCC6L; NUF2; ANLN; CEP152; * *KIFC3; MAP1LC3B; BRSK2; SPEG2; KRT14; ARC*
DNA damage response	*MCM10; RAD51; ANKRD32 (SLF1)*		*RAD51AP1; ERCC6L; DNA2; ARHGAP11A*
Drug metabolism		*CYB5R2; SLC47A1; * *FMO3*	
Extracellular matrix	*COL7A1; KRTAP3-2*	*ECM2; * *COL9A3*	
Glycosylation		*GALNT18; * *GALC; AMY1C*	*FUT3*
Growth factors		*IGFBP2; ANGPTL2; ISM1; EDA; * *IGFALS*	*AREG*
Growth factors receptors	*RHBDF1*	*NOTCH2; ROR1; * *EFNA1*	
Hypoxia response			*EGLN3*
Inflammation and immunity	*IL18;* * SPON2*		*CD8B;* * NCF2; SLAMF7*
Intracellular transport	*SPIRE2; BAIAP3; TUBB2A; SYT13*	*BET1L;* * C16orf62; LMF1; MYRIP*	*LPHN2;* * KIFC3; RAB37; GOLGA7B*
Ion transport	*PLLP; MAGED2; SLC4A11*	*KCNS3; CNKSR3; * *STAC3; ATP6AP1L;*	*STOM*
Lipid metabolism		*CYB5R2; CROT; * *LMF1; FABP6*	*ELOVL2;* * SLCO1B3*
Lysosomal enzymes	*IDS*		
Mucosa protection			*MUC6*
Phosphatases	*PTPRN2*	*SGPP2*	*PTPN13*
Proteolysis		*TPP2*	*ADAM30;* * SERPINA3*
RNA maturation	*SRPK3*	*SNAR-G1; SNAR-F; SNAR-G2; SNAR-H; SNAR-D; SNAR-A3*	
Signal transduction	*PLCB1; DDR1; NXN; RNF157*	*DLC1; SGPP2; STK32C; * *KSR2*	*ATRNL1; DKK4; ARHGAP11A; * *ADRB1; RAB37; ADRB2; TBC1D4*
Stress response		*HSPA1A*	
Transcription	*MAF; MTA2; * *ZNF276; NXN; ZNF83*	*ZNF316; LEF1; TFAP2C; * *KLF12; HBP1; ZFP62*	*ZSCAN20* *; ZNF462*
Transporters	*SLC43A3;* * ABCC3; AKR1C1*	*SLC47A1; XK; SLC2A8*	*SLC2A6;* * SLC7A2*
Ubiquitin proteasome pathway	*RNF157*	*TPP2;* * RNF130; OTUD1*	

Only genes showing a fold change “Mean B4GALNT2/Mean Neo” ≥2, a *p* value ≤ 0.05 and a level of expression either in Neo or in B4GALNT2 ≥ 50 are reported. Genes up-regulated or down-regulated are marked in red or blue, respectively.

**Table 5:** Genes consistently modulated by B4GALNT2 expression in SW480, SW620 and LS174T.

Cell Line	Type of Transfection	Gene Symbol						
		*MCOLN2*	*PLLP*	*FILIP1L*	*FAM231A*	*SPON2*	*COL20A1*	*BCL2L10*
SW480	Neo	10	674	59	8	10,476	40	26
	B4GALNT2	22	441	36	5	5779	21	12
SW620	Neo	19	803	107	16	8579	54	33
	B4GALNT2	59	509	57	8	5447	28	12
**LS174T**	Neo	4	104	20	24	338	14	8
	B4GALNT2	9	52	13	15	181	8	2
	Gene name	Mucolipin 2	Plasmolipin	Filamin A interacting protein like	Family with sequence similarity 231 member A	Spondin 2	Collagen XX α1	BCL2-like 10
	Role in cancer	Promotes glioma progression	No information	Inhibits CRC progression	No information	Promotes malignancy of CRC	Overexpressed in glioma	Tumor suppressor
	PMID	27248469		7750216		26686083	31556357	31894274 27770580

Numbers indicate the expression level of the seven genes in the six cell lines, which were the only showing significantly (*p* ≤ 0.05) different expression level in B4GALNT2-transfected cells, compared with Neo. Transcriptomic analysis of LS174T cells has been published in Ref. [27]. The role of the genes in cancer was deduced from a PubMed search. The relevant papers have been indicated with their PubMed identification numbers (PMID).

**Table 6:** Summary of the phenotypic changes induced by FUT6 or B4GALNT2 in SW480, SW620 and LS174T cell lines.

Biological Function	SW480		SW620		LS174T *
	FUT6	B4GALNT2	FUT6	B4GALNT2	B4GALNT2
Proliferation rate	Unchanged	Unchanged	Unchanged	Down	Unchanged
Clonogenic ability (solid)	Up	Down	Up	Down	Unchanged
Soft agar growth	Unchanged	Down	Up	Down	Down
Spheroid formation	Unchanged	Down	Unchanged	Down	Down
Wound healing ability	Unchanged	Unchanged	Up	Down	Unchanged
ALDH expression	Down	Down	Unchanged	Down	Down

* From Ref. 27. LS174T cells express constitutively high levels of FUT6/sLe^x^ and have not been transfected with FUT6.

## Supplementary Materials

The following are available online at https://www.mdpi.com/1422-0067/21/18/6558/s1, Figure S1: Biochemical characterization of FUT6 or B4GALNT2-transfected cell lines, Table S1: Genes up- or down-regulated in both SW480 and SW620 cells in response to FUT6 expression, Table S2: Genes up- or down-regulated in both SW480 and SW620 cells in response to B4GALNT2 expression, Table S3: Genes consistently modulated by expression of either glycosyltransferase.

## Abbreviations

ALDH	aldheyde dehydrogenase 1
ANOVA	Analysis of variance
CRC	Colorectal cancer
DEAB	N,*N*-diethylaminobenzaldehyde
DT	Doubling time
PBS	Phosphate buffered saline
TCGA	The Cancer Genome Atlas

## References

[1] Dall’Olio F., Malagolini N., Trinchera M., Chiricolo M. (2012). Mechanisms of cancer-associated glycosylation changes. Front. Biosci..

[2] Dall’Olio F., Malagolini N., Chiricolo M. (2012). Glycosylation in cancer. Spec. Period. Rep. Carbohydr. Chem..

[3] Gomes Ferreira I., Pucci M., Venturi G., Malagolini N., Chiricolo M., Dall’Olio F. (2018). Glycosylation as a Main Regulator of Growth and Death Factor Receptors Signaling. Int. J. Mol. Sci..

[4] Pinho S.S., Reis C.A. (2015). Glycosylation in cancer: Mechanisms and clinical implications. Nat. Rev. Cancer.

[5] Nakamori S., Kameyama M., Imaoka S., Furukawa H., Ishikawa O., Sasaki Y., Kabuto T., Iwanaga T., Matsushita Y., Irimura T. (1993). Increased expression of sialyl Lewisx antigen correlates with poor survival in patients with colorectal carcinoma: Clinicopathological and immunohistochemical study. Cancer Res..

[6] Nakamori S., Kameyama M., Imaoka S., Furukawa H., Ishikawa O., Sasaki Y., Izumi Y., Irimura T. (1997). Involvement of carbohydrate antigen sialyl Lewisx in colorectal cancer metastasis. Dis. Colon Rectum.

[7] Trinchera M., Aronica A., Dall’Olio F. (2017). Selectin Ligands Sialyl-Lewis a and Sialyl-Lewis x in Gastrointestinal Cancers. Biology.

[8] Yamada N., Chung Y.S., Maeda K., Sawada T., Ikehara T., Nishino H., Okuno M., Sowa M. (1995). Increased expression of sialyl Lewis A and sialyl Lewis X in liver metastases of human colorectal carcinoma. Invasion Metastasis.

[9] Carvalho A.S., Harduin-Lepers A., Magalhaes A., Machado E., Mendes N., Costa L.T., Matthiesen R., Almeida R., Costa J., Reis C.A. (2010). Differential expression of a-2,3-sialyltransferases and a-1,3/4-fucosyltransferases regulates the levels of sialyl Lewis a and sialyl Lewis x in gastrointestinal carcinoma cells. Int. J. Biochem. Cell Biol..

[10] Ito H., Hiraiwa N., Sawada-Kasugai M., Akamatsu S., Tachikawa T., Kasai Y., Akiyama S., Ito K., Takagi H., Kannagi R. (1997). Altered mRNA expression of specific molecular species of fucosyl- and sialyl-transferases in human colorectal cancer tissues. Int. J. Cancer.

[11] Izawa M., Kumamoto K., Mitsuoka C., Kanamori C., Kanamori A., Ohmori K., Ishida H., Nakamura S., Kurata-Miura K., Sasaki K. (2000). Expression of sialyl 6-sulfo Lewis X is inversely correlated with conventional sialyl Lewis X expression in human colorectal cancer. Cancer Res..

[12] Miyazaki K., Ohmori K., Izawa M., Koike T., Kumamoto K., Furukawa K., Ando T., Kiso M., Yamaji T., Hashimoto Y. (2004). Loss of disialyl Lewisa the ligand for lymphocyte inhibitory receptor sialic acid-binding immunoglobulin-like lectin-7 (Siglec-7) associated with increased sialyl Lewis a expression on human colon cancers. Cancer Res..

[13] Trinchera M., Malagolini N., Chiricolo M., Santini D., Minni F., Caretti A., Dall’Olio F. (2011). The biosynthesis of the selectin-ligand sialyl Lewis x in colorectal cancer tissues is regulated by fucosyltransferase VI and can be inhibited by an RNA interference-based approach. Int. J. Biochem. Cell Biol..

[14] Serafini-Cessi F., Dall’Olio F. (1983). Guinea-pig kidney b-N-acetylgalactosaminyltransferase towards Tamm-Horsfall glycoprotein. Requirement of sialic acid in the acceptor for transferase activity. Biochem. J..

[15] Lo Presti L., Cabuy E., Chiricolo M., Dall’Olio F. (2003). Molecular Cloning of the Human b1,4 N-acetylgalactosaminyltransferase responsible for the biosynthesis of the Sda histo-blood group antigen: The sequence predicts a very long cytoplasmic domain. J. Biochem..

[16] Montiel M.D., Krzewinski-Recchi M.A., Delannoy P., Harduin-Lepers A. (2003). Molecular cloning, gene organization and expression of the human UDP-GalNAc:Neu5Aca2-3Galb-R b1,4-N-acetylgalactosaminyltransferase responsible for the biosynthesis of the blood group Sda/Cad antigen: Evidence for an unusual extended cytoplasmic domain. Biochem. J..

[17] Smith P.L., Lowe J.B. (1994). Molecular cloning of a murine N-acetylgalactosamine transferase cDNA that determines expression of the T lymphocyte-specific CT oligosaccharide differentiation antigen. J. Biol. Chem..

[18] Dall’Olio F., Malagolini N., Chiricolo M., Trinchera M., Harduin-Lepers A. (2014). The expanding roles of the Sda/Cad carbohydrate antigen and its cognate glycosyltransferase B4GALNT2. Biochim. Biophys. Acta.

[19] Stenfelt L., Hellberg A., Moller M., Thornton N., Larson G., Olsson M.L. (2019). Missense mutations in the C-terminal portion of the B4GALNT2-encoded glycosyltransferase underlying the Sd(a-) phenotype. Biochem. Biophys. Rep..

[20] Groux-Degroote S., Schulz C., Cogez V., Noel M., Portier L., Vicogne D., Solorzano C., Dall’Olio F., Steenackers A., Mortuaire M. (2018). The extended cytoplasmic tail of the human B4GALNT2 is critical for its Golgi targeting and post-Golgi sorting. FEBS J..

[21] Dohi T., Yuyama Y., Natori Y., Smith P.L., Lowe J.B., Oshima M. (1996). Detection of N-acetylgalactosaminyltransferase mRNA which determines expression of Sda blood group carbohydrate structure in human gastrointestinal mucosa and cancer. Int. J. Cancer.

[22] Malagolini N., Dall’Olio F., Di Stefano G., Minni F., Marrano D., Serafini-Cessi F. (1989). Expression of UDP-GalNAc:NeuAc a2,3Gal b-R beta 1,4(GalNAc to Gal) N-acetylgalactosaminyltransferase involved in the synthesis of Sda antigen in human large intestine and colorectal carcinomas. Cancer Res..

[23] Malagolini N., Santini D., Chiricolo M., Dall’Olio F. (2007). Biosynthesis and expression of the Sda and sialyl Lewis x antigens in normal and cancer colon. Glycobiology.

[24] Groux-Degroote S., Wavelet C., Krzewinski-Recchi M.A., Portier L., Mortuaire M., Mihalache A., Trinchera M., Delannoy P., Malagolini N., Chiricolo M. (2014). B4GALNT2 gene expression controls the biosynthesis of Sda and sialyl Lewis X antigens in healthy and cancer human gastrointestinal tract. Int. J. Biochem. Cell Biol..

[25] Kawamura Y.I., Toyota M., Kawashima R., Hagiwara T., Suzuki H., Imai K., Shinomura Y., Tokino T., Kannagi R., Dohi T. (2008). DNA hypermethylation contributes to incomplete synthesis of carbohydrate determinants in gastrointestinal cancer. Gastroenterology.

[26] Wang H.R., Hsieh C.Y., Twu Y.C., Yu L.C. (2008). Expression of the human Sda b-1,4-N-acetylgalactosaminyltransferase II gene is dependent on the promoter methylation status. Glycobiology.

[27] Pucci M., Gomes Ferreira I., Orlandani M., Malagolini N., Ferracin M., Dall’Olio F. (2020). High expression of the Sda synthase B4GALNT2 associates with good prognosis and attenuates stemness in colon cancer. Cells.

[28] Kawamura Y.I., Kawashima R., Fukunaga R., Hirai K., Toyama-Sorimachi N., Tokuhara M., Shimizu T., Dohi T. (2005). Introduction of Sda carbohydrate antigen in gastrointestinal cancer cells eliminates selectin ligands and inhibits metastasis. Cancer Res..

[29] Kawamura Y.I., Adachi Y., Curiel D.T., Kawashima R., Kannagi R., Nishimoto N., Dohi T. (2014). Therapeutic adenoviral gene transfer of a glycosyltransferase for prevention of peritoneal dissemination and metastasis of gastric cancer. Cancer Gene Ther..

[30] Leibovitz A., Stinson J.C., McCombs W.B., McCoy C.E., Mazur K.C., Mabry N.D. (1976). Classification of human colorectal adenocarcinoma cell lines. Cancer Res..

[31] Hiller K.M., Mayben J.P., Bendt K.M., Manousos G.A., Senger K., Cameron H.S., Weston B.W. (2000). Transfection of a1,3 fucosyltransferase antisense sequences impairs the proliferative and tumorigenic ability of human colon carcinoma cells. Mol. Carcinog..

[32] Hirakawa M., Takimoto R., Tamura F., Yoshida M., Ono M., Murase K., Sato Y., Osuga T., Sato T., Iyama S. (2014). Fucosylated TGF-b receptors transduces a signal for epithelial-mesenchymal transition in colorectal cancer cells. Br. J. Cancer.

[33] Liang L., Gao C., Li Y., Sun M., Xu J., Li H., Jia L., Zhao Y. (2017). miR-125a-3p/FUT5-FUT6 axis mediates colorectal cancer cell proliferation, migration, invasion and pathological angiogenesis via PI3K-Akt pathway. Cell Death Dis..

[34] Pan S., Liu Y., Liu Q., Xiao Y., Liu B., Ren X., Qi X., Zhou H., Zeng C., Jia L. (2019). HOTAIR/miR-326/FUT6 axis facilitates colorectal cancer progression through regulating fucosylation of CD44 via PI3K/AKT/mTOR pathway. Biochim. Biophys. Acta Mol. Cell Res..

[35] Hanley W.D., Burdick M.M., Konstantopoulos K., Sackstein R. (2005). CD44 on LS174T colon carcinoma cells possesses E-selectin ligand activity. Cancer Res..

[36] Schmid F., Wang Q., Huska M.R., Andrade-Navarro M.A., Lemm M., Fichtner I., Dahlmann M., Kobelt D., Walther W., Smith J. (2016). SPON2, a newly identified target gene of MACC1, drives colorectal cancer metastasis in mice and is prognostic for colorectal cancer patient survival. Oncogene.

[37] Venturi G., Gomes F.I., Pucci M., Ferracin M., Malagolini N., Chiricolo M., Dall’Olio F. (2019). Impact of sialyltransferase ST6GAL1 overexpression on different colon cancer cell types. Glycobiology.

[38] Ferracin M., Bassi C., Pedriali M., Pagotto S., D’Abundo L., Zagatti B., Corra F., Musa G., Callegari E., Lupini L. (2013). miR-125b targets erythropoietin and its receptor and their expression correlates with metastatic potential and ERBB2/HER2 expression. Mol. Cancer.

