# Supplementary material for: The Sda Synthase B4GALNT2 Reduces Malignancy and Stemness in Colon Cancer Cell Lines Independently of Sialyl Lewis X Inhibition
Source: Int J Mol Sci. 2020 Sep 8;21(18):6558. doi: 10.3390/ijms21186558 (PMC7555213; doi:10.3390/ijms21186558)
Supplement: Supplementary file 1 [file ijms-21-06558-s001.zip › Fig. S1.pptx]

## Slide 1
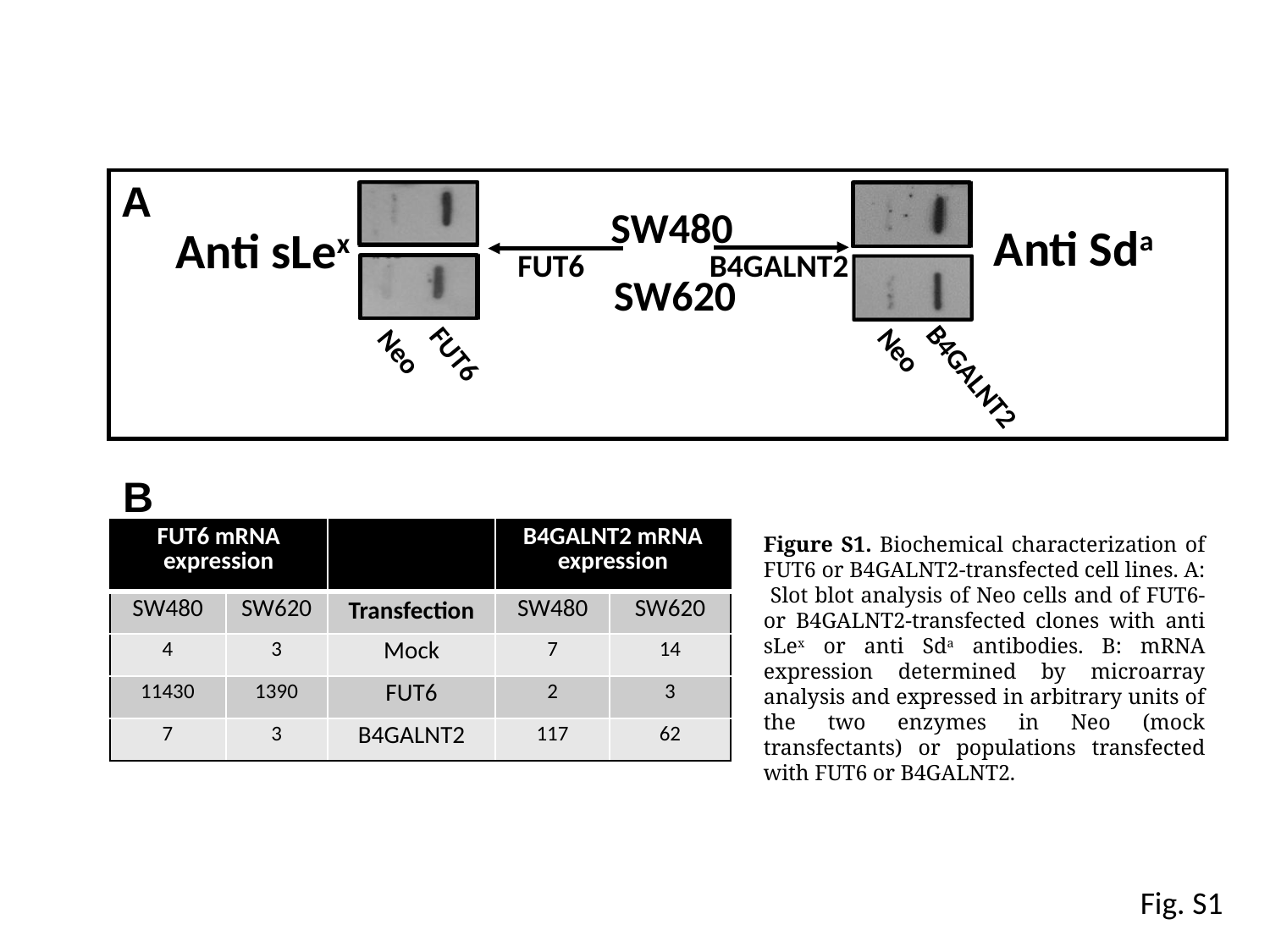

A
SW480
Anti Sda
Anti sLex
FUT6
B4GALNT2
SW620
Neo
Neo
FUT6
B4GALNT2
B
| FUT6 mRNA expression | | | B4GALNT2 mRNA expression | |
| --- | --- | --- | --- | --- |
| SW480 | SW620 | Transfection | SW480 | SW620 |
| 4 | 3 | Mock | 7 | 14 |
| 11430 | 1390 | FUT6 | 2 | 3 |
| 7 | 3 | B4GALNT2 | 117 | 62 |
Figure S1. Biochemical characterization of FUT6 or B4GALNT2-transfected cell lines. A: Slot blot analysis of Neo cells and of FUT6- or B4GALNT2-transfected clones with anti sLex or anti Sda antibodies. B: mRNA expression determined by microarray analysis and expressed in arbitrary units of the two enzymes in Neo (mock transfectants) or populations transfected with FUT6 or B4GALNT2.
Fig. S1
