# Supplementary material for: The Sda Synthase B4GALNT2 Reduces Malignancy and Stemness in Colon Cancer Cell Lines Independently of Sialyl Lewis X Inhibition
Source: Int J Mol Sci. 2020 Sep 8;21(18):6558. doi: 10.3390/ijms21186558 (PMC7555213; doi:10.3390/ijms21186558)
Supplement: Supplementary file 1 [file ijms-21-06558-s001.zip › Table S1.docx]

Supplementary Table 1. Genes up- or down-regulated in both SW480 and SW620 cells in response to FUT6 expression

| **Gene symbol** | **Mean Neo** | **Mean FUT6** | **Gene Name** | **Function** |
| --- | --- | --- | --- | --- |
| *KCNJ2* | 14.1 | 54.3 | potassium channel, inwardly rectifying subfamily J, member 2 | Allows potassium to flow into the cell |
| *GPAT2* | 42.8 | 158.6 | glycerol-3-phosphate acyltransferase 2, mitochondrial | Involved in processing step during piRNA biosynthesis. |
| *CPLX2* | 19.3 | 68.9 | complexin 2 | Positively regulates a late step in exocytosis of various cytoplasmic vesicles |
| *APOH* | 57.6 | 185.4 | apolipoprotein H (beta-2-glycoprotein I) | Binds to various kinds of negatively charged substances such as heparin, phospholipids, and dextran sulfate |
| *SNORA30* | 90.3 | 284.3 | small nucleolar RNA, H/ACA box 30 | Small nucelolar RNA |
| *OXR1* | 143.2 | 446.1 | oxidation resistance 1 | May be involved in protection from oxidative damage |
| *MYH7B* | 3383.7 | 10495.5 | myosin, heavy chain 7B, cardiac muscle, beta | Involved in muscle contraction |
| *CENPI* | 27.3 | 84.3 | centromere protein I | Involved in accurate chromosome alignment and segregation |
| *CAPN15* | 254.5 | 767.0 | calpain 15 | May function as a transcription factor |
| *SNORA62* | 272.5 | 815.1 | small nucleolar RNA, H/ACA box 62 | Small nucelolar RNA |
| *CD55* | 338.7 | 114.6 | CD55 molecule, decay accelerating factor for complement (Cromer blood group) | Inhibits complement activation |
| *FXYD4* | 54.0 | 18.1 | FXYD domain containing ion transport regulator 4 | Modulates the properties of the Na,K-ATPase |
| *BRSK2* | 93.3 | 31.0 | BR serine/threonine kinase 2 | Plays a role in the regulation of the mitotic cell cycle progress and the onset of mitosis. Regulates reorganization of the actin cytoskeleton |
| *TMEM255B* | 274.5 | 89.8 | transmembrane protein 255B | Little or no information |
| *CFAP70* | 161.0 | 52.2 | cilia and flagella associated protein 70 | Little or no information |
| *XDH* | 156.0 | 50.5 | xanthine dehydrogenase | Key enzyme in purine degradation |
| *MCAM* | 1107.7 | 355.3 | melanoma cell adhesion molecule | Plays a role in cell adhesion |
| *CXCL8* | 231.1 | 73.8 | chemokine (C-X-C motif) ligand 8 | Chemotactic factor |
| *C11orf96* | 5360.3 | 1701.6 | chromosome 11 open reading frame 96 | Little or no information |
| *NCF2* | 130.0 | 41.2 | neutrophil cytosolic factor 2 | Subunit of the NADPH oxidase complex found in neutrophils, which produces superoxide to kill bacteria |
| *RASGEF1A* | 128.6 | 40.7 | RasGEF domain family, member 1A | Guanine nucleotide exchange factor specific for RAS |
| *FAM228B* | 347.8 | 109.9 | family with sequence similarity 228, member B | Little or no information |
| *CAPN5* | 1312.9 | 413.2 | calpain 5 | Calcium-dependent cysteine protease involved in signal transduction |
| *SLIT1* | 60.3 | 18.8 | slit homolog 1 (Drosophila) | Acts as molecular guidance cue in cellular migration |
| *BEX2* | 9397.4 | 2922.5 | brain expressed X-linked 2 | Regulator of mitochondrial apoptosis and G1 cell cycle. Regulates transcription. Tumor suppressor. |
| *WDR78* | 53.2 | 16.5 | WD repeat domain 78 | Little or no information |
| *CAPN8* | 72.3 | 22.2 | calpain 8 | Involved in membrane trafficking in mucus cells |
| *SCEL* | 1132.9 | 343.4 | sciellin | May function in the assembly or regulation of proteins in the cornified envelope |
| *PTPN13* | 109.7 | 33.2 | protein tyrosine phosphatase, non-receptor type 13 (APO-1/CD95 (Fas)-associated phosphatase) | Tyrosine phosphatase which regulates negatively FAS-induced apoptosis |
| *TPSAB1* | 63.6 | 18.8 | tryptase alpha/beta 1 | Tryptases are trypsin-like serine proteases |
| *BHLHE41* | 489.9 | 143.2 | basic helix-loop-helix family, member e41 | Transcriptional repressor involved in the regulation of the circadian rhythm |
| *FILIP1* | 58.2 | 16.9 | filamin A interacting protein 1 | By acting through a filamin-A/F-actin axis, it controls the start of neocortical cell migration |
| *REPS2* | 59.3 | 17.0 | RALBP1 associated Eps domain containing 2 | Involved in growth factor signaling |
| *GRB10* | 1072.8 | 304.4 | growth factor receptor-bound protein 10 | Binds to insulin and insulin like growth-factor receptors, inhibiting signaling |
| *GBP3* | 84.3 | 23.8 | guanylate binding protein 3 | Encodes a member of the guanylate-binding protein (GBP) family |
| *TMEM159* | 512.0 | 137.6 | transmembrane protein 159 | Little or no information |
| *HRK* | 708.6 | 188.6 | harakiri, BCL2 interacting protein | Promotes apoptosis by interacting with the apoptotic inhibitors BCL-2 and BCL-X(L) |
| *DIP2C* | 149.4 | 39.4 | DIP2 disco-interacting protein 2 homolog C (Drosophila) | May be a transcription factor binding |
| *SERPINE2* | 942.6 | 244.0 | serpin peptidase inhibitor, clade E (nexin, plasminogen activator inhibitor type 1), member 2 | Inhibits serine proteases |
| *CPLX1* | 234.5 | 59.5 | complexin 1 | Positively regulates a late step in exocytosis of various cytoplasmic vesicles |
| *TFPI* | 58.0 | 14.4 | tissue factor pathway inhibitor (lipoprotein-associated coagulation inhibitor) | Serine protease inhibitor that regulates the tissue factor (TF)-dependent pathway of blood coagulation |
| *ADRBK2* | 208.1 | 51.5 | adrenergic, beta, receptor kinase 2 | Phosphorylates the agonist-occupied form of the β-adrenergic receptor |
| *ZIC5* | 54.9 | 13.5 | Zic family member 5 | May act as a transcriptional repressor |
| *MIA* | 75.7 | 18.5 | melanoma inhibitory activity | Growth inhibitor |
| *EPAS1* | 99.7 | 24.1 | endothelial PAS domain protein 1 | Transcription factor involved in the induction of oxygen regulated genes |
| *PEAR1* | 57.1 | 13.4 | platelet endothelial aggregation receptor 1 | Platelet receptor that signals upon the formation of platelet-platelet contacts |
| *CREB5* | 102.7 | 23.9 | cAMP responsive element binding protein 5 | Binds to the cAMP response element and activates transcription |
| *AHNAK2* | 98.3 | 22.9 | AHNAK nucleoprotein 2 | May play a role in calcium signaling by associating with calcium channel proteins |
| *ZNF462* | 125.8 | 28.7 | zinc finger protein 462 | May be involved in transcriptional regulation |
| *C16orf45* | 198.0 | 43.5 | chromosome 16 open reading frame 45 | Little or no information |
| *AKAP12* | 1432.1 | 303.7 | A kinase (PRKA) anchor protein 12 | Associates with protein kinases and phosphatase, serving as a scaffold protein in signal transduction |
| *PRDM13* | 177.8 | 36.3 | PR domain containing 13 | Little or no information |
| *BEST1* | 177.2 | 34.8 | bestrophin 1 | Forms calcium-sensitive chloride channels |
| *NTN4* | 58.7 | 11.5 | netrin 4 | Netrins are laminin-related proteins |
| *GPR126* | 65.2 | 12.4 | G protein-coupled receptor 126 | G-protein coupled receptor which is activated by type IV collagen |
| *ANTXR2* | 175.4 | 32.6 | anthrax toxin receptor 2 | Necessary for cellular interactions with laminin and the extracellular matrix |
| *TIMP3* | 138.5 | 25.4 | TIMP metallopeptidase inhibitor 3 | Inactivates metalloproteases |
| *TUBB2B* | 1559.3 | 247.2 | tubulin, beta 2B class IIb | Major constituent of microtubules |
| *TRPV6* | 212.5 | 31.5 | transient receptor potential cation channel, subfamily V, member 6 | Mediates Ca(2+) uptake in various tissues, including the intestine |
| *HES7* | 84.9 | 10.4 | hes family bHLH transcription factor 7 | Transcriptional repressor |
| *HS3ST1* | 286.8 | 32.5 | heparan sulfate (glucosamine) 3-O-sulfotransferase 1 | Involved in heparan sulfate biosynthesis |
| *CALB2* | 130.7 | 14.4 | calbindin 2 | Calcium binding |
| *CALCA* | 52.6 | 2.2 | calcitonin-related polypeptide alpha | Calcitonin and related receptors are a family of G-protein-coupled receptors |

“Mean Neo” and “Mean FUT6” represent the mean expression value of SW480 and SW620 Neo- and FUT6-expressing cells respectively. Here, we reported only protein coding genes showing a fold change “Mean FUT6 / Mean Neo” ≥3, a corrected p value ≤ 0.05 and a level of expression either in Neo or in FUT6 ≥ 50. Up-regulated and down-regulated genes are marked in red and blue, respectively. Information on gene function were deduced from https://www.genecards.org/.
