# Supplementary material for: The Sda Synthase B4GALNT2 Reduces Malignancy and Stemness in Colon Cancer Cell Lines Independently of Sialyl Lewis X Inhibition
Source: Int J Mol Sci. 2020 Sep 8;21(18):6558. doi: 10.3390/ijms21186558 (PMC7555213; doi:10.3390/ijms21186558)
Supplement: Supplementary file 1 [file ijms-21-06558-s001.zip › Table S2.docx]

Supplementary Table 2. Genes up- or down-regulated in both SW480 and SW620 cells in response to B4GALNT2 expression.

| **Gene symbol** | **Mean Neo** | **Mean B4GALNT2** | **Gene name** | **Function** |
| --- | --- | --- | --- | --- |
| *G0S* | 785 | 1603 | G0/G1 switch 2 | Promotes apoptosis by preventing the formation of protective BCL2-BAX heterodimers |
| *ORC6* | 468 | 898 | Origin recognition complex, subunit 6 | Coordinates chromosome replication and segregation with cytokinesis |
| *CDCA5* | 7025 | 12027 | Cell division cycle associated 5 | Regulator of sister chromatid cohesion in mitosis |
| *MCM10* | 268 | 457 | Minichromosome maintenance complex component 10 | Acts as a DNA replication initiation factor. Prevents DNA from damage during replication |
| *MAF* | 55 | 91 | V-maf avian musculoaponeurotic fibrosarcoma oncogene homolog | Can be a transcriptional activator or repressor. Behaves as an oncogene or a tumor suppressor |
| *PPIH* | 3920 | 6365 | Peptidylprolyl isomerase H (cyclophilin H) | Assists protein folding |
| *RAD51* | 2049 | 3381 | RAD51 recombinase | Involved in DNA repair through homologous recombination |
| *ANKRD32 (SLF1)* | 354 | 575 | Ankyrin repeat domain 32 | Involved in the DNA damage response and genomic stability maintenance |
| *SLC43A3* | 1721 | 2788 | Solute carrier family 43, member 3 | Putative transporter |
| *IL18* | 532 | 829 | Interleukin 18 | Proinflammatory cytokine |
| *MTA2* | 1835 | 2889 | Metastasis associated 1 family, member 2 | Involved in transcription regulation as repressor and activator, interacting with histones |
| *ZNF276* | 138 | 90 | Zinc finger protein 276 | May be involved in transcriptional regulation. |
| *DDR1* | 1881 | 1228 | Discoidin domain receptor tyrosine kinase 1 | Receptor tyrosine kinase acting as a cell surface adhesion molecule, regulating migration and proliferation |
| *PLLP* | 736 | 471 | Plasmolipin | Could participate in ion transport events |
| *IDS* | 373 | 236 | Iduronate 2-sulfatase | Lysosomal enzyme involved in the degradation of dermatan- and heparan sulfate |
| *SPIRE2* | 1942 | 1243 | Spire-type actin nucleation factor 2 | Actin nucleation factor involved in intracellular vesicle transport and for asymmetric cell division during meiosis |
| *NXN* | 358 | 232 | Nucleoredoxin | Functions as a redox-dependent negative regulator of Wnt signaling and as a transcriptional regulator. |
| *MAGED2* | 4889 | 2894 | Melanoma antigen family D, 2 | Regulates NaCl co-transporters |
| *LGALS7* | 1245 | 740 | Lectin, galactoside-binding, soluble, 7 | Pro-apoptotic galectin |
| *SPON2* | 9495 | 5535 | Spondin 2, extracellular matrix protein | Functions as an opsonin for macrophage phagocytosis of bacteria. |
| *ZNF83* | 80 | 47 | Zinc finger protein 83 | May be involved in transcriptional regulation |
| *RNF157* | 66 | 38 | Ring finger protein 157 | Ubiquitin ligase preventing apoptosis. Acts as a downstream effector of the PI3K and MAPK signaling |
| *FILIP1L* | 81 | 45 | Filamin A interacting protein 1-like | When overexpressed in endothelial cells, leads to inhibition of cell proliferation and migration and an increase in apoptosis. |
| *SRPK3* | 138 | 78 | SRSF protein kinase 3 | Phosphorylates the SR splicing factor SRSF1 |
| *ABCC3* | 2539 | 1440 | ATP-binding cassette, sub-family C (CFTR/MRP), member 3 | May act as an inducible transporter in the biliary and intestinal excretion of organic anions |
| *RHBDF1* | 1118 | 626 | Rhomboid 5 homolog 1 (Drosophila) | Regulates ADAM17 protease, releasing epidermal growth factor (EGF) receptor ligands and TNF |
| *PTPRN2* | 4125 | 2379 | Protein tyrosine phosphatase, receptor type, N polypeptide 2 | Regulates PI(4,5)P2 level in the plasma membrane and actin dynamics related to cell migration and metastasis |
| *BAIAP3* | 191 | 103 | BAI1-associated protein 3 | Functions in endosome to Golgi retrograde transport. |
| *SLC4A11* | 1517 | 740 | Solute carrier family 4, sodium borate transporter, member 11 | Sodium-coupled borate cotransporter that is essential for borate homeostasis, |
| *SEMA3B* | 8740 | 4612 | Sema domain, immunoglobulin domain (Ig), short basic domain, secreted, (semaphorin) 3B | Inhibits axonal extension and acts as a tumor suppressor by inducing apoptosis |
| *TUBB2A* | 6276 | 3199 | Tubulin, beta 2A class IIa | Component of microtubules, key participants in processes such as mitosis and intracellular transport |
| *AKR1C1* | 721 | 360 | Aldo-keto reductase family 1, member C1 | In the liver and intestine, it may have a role in the transport of bile |
| *COL7A1* | 299 | 142 | Collagen, type VII, alpha 1 | May contribute to epithelial basement membrane organization and adherence |
| *SYT13* | 1037 | 497 | Synaptotagmin XIII | May be involved in transport vesicle docking to the plasma membrane |
| *KRTAP3-2* | 220 | 89 | Keratin associated protein 3-2 | Member of the keratin-associated protein (KAP) family |
| *KRT15* | 1421 | 610 | Keratin 15, type I | Component of the intermediate filaments |
| *SPTBN5* | 250 | 99 | Spectrin, beta, non-erythrocytic 5 | Binds actin and kinesin |
| *EMP1* | 16043 | 6672 | Epithelial membrane protein 1 | Little or no information |
| *RGCC* | 842 | 328 | Regulator of cell cycle | Overexpression activates or suppresses cell cycle progression |
| *CDHR2* | 322 | 132 | Cadherin-related family member 2 | Involved in cell-cell adhesion and contact inhibition in epithelial cells. Candidate tumor suppressor |
| *PLCB1* | 536 | 227 | Phospholipase C, beta 1 (phosphoinositide-specific) | Produces the second messenger molecules diacylglycerol (DAG) and inositol 1,4,5-trisphosphate (IP3) |
| *IQCH* | 68 | 24 | IQ motif containing H | May play a regulatory role in spermatogenesis |
| *DISC1* | 119 | 35 | Disrupted in schizophrenia 1 | Positively regulates Wnt-mediated proliferation. Plays a role in the microtubule network formation |
| *CDIP1* | 52 | 15 | Cell death-inducing p53 target 1 | Acts as an important p53-apoptotic effector |
| *CD44* | 475 | 108 | CD44 molecule (Indian blood group) | Receptor for hyaluronic acid and other ligands |

“Mean Neo” and “Mean B4GALNT2” represent the mean expression value of SW480 and SW620 Neo- and B4GALNT2-expressing cells respectively. Here, we reported only genes showing a fold change “Mean B4GALNT2 / Mean Neo” ≥2, a *p* value ≤ 0.05 and a level of expression either in Neo or in B4GALNT2 ≥ 50. Up-regulated and down-regulated genes are marked in red and blue, respectively. Information on gene function were deduced from https://www.genecards.org/.
