# Supplementary material for: The Sda Synthase B4GALNT2 Reduces Malignancy and Stemness in Colon Cancer Cell Lines Independently of Sialyl Lewis X Inhibition
Source: Int J Mol Sci. 2020 Sep 8;21(18):6558. doi: 10.3390/ijms21186558 (PMC7555213; doi:10.3390/ijms21186558)
Supplement: Supplementary file 1 [file ijms-21-06558-s001.zip › Table S3.docx]

Supplementary Table 3. Genes consistently modulated by expression of either glycosyltransferase

| **Gene** | **Function** | **Functional class** | **Cell line** | **Regulation** |
| --- | --- | --- | --- | --- |
| *ANLN* | Actin-binding protein required for cytokinesis | Cytoskeleton-cytokinesis | SW620 | Up |
| *RAD51AP1* | Participates to homologous recombination repair | DNA damage response | SW620 | Up |
| *CYB5R2* | Involved in desaturation and elongation of fatty acids, cholesterol biosynthesis, drug metabolism | Drug metabolism | SW480 | Up |
| *COL9A3* | Structural component of hyaline cartilage | Extracellular matrix | SW480 | Down |
| *IGFBP2* | Binds to IGF, prolonging its activity | Growth factors | SW480 | Up |
| *SGPP2* | Degrades the bioactive signaling molecule sphingosine 1-phosphate | Phosphatases | SW480 | Up |
| *PLCB1* | Production of the second messenger molecules diacylglycerol (DAG) and inositol 1,4,5-trisphosphate (IP3) | Signal transduction | SW480 | Down |
| *LEF1* | Transcription factor of the Wnt signaling, activates *MYC* and *CCND1* expression and enhances proliferation of pancreatic tumor cells | Transcription | SW480 | Up |
| *ABCC3* | May act as an inducible transporter in the biliary and intestinal excretion of organic anions | Transporters | SW480 | Down |
| *OTUD1* | Removes ubiquitin | Ubiquitin proteasome pathway | SW480 | Down |

Information on the gene function was deduced from the Gene Cards web site (https://www.genecards.org/).
